# The public health ethics and economics of prioritizing prevention and equity

**DOI:** 10.1093/pubmed/fdaf138

**Published:** 2025-11-18

**Authors:** Kathryn Hamilton, Frances C Butcher, Jane Powell

**Affiliations:** North Bristol NHS Trust and University Hospitals Bristol and Weston NHS Foundation Trust, Bristol, BS10 5NB, UK; Ethox Centre, Nuffield Department of Population Health, University of Oxford, OX3 7LF, UK; University of the West of England, BS16 1QY, UK

**Keywords:** economics, ethics, population-based and preventative services

## Abstract

Internationally there are significant challenges to improving and protecting population health through effective prevention. In this novel analysis, we integrate public health evidence, ethics, and economics to argue that current health resourcing strategies neglect prevention, underestimate the social determinants of health, and fail to engage with the ethical dimensions of economic decision-making. We highlight the risk that overreliance on economic evidence may marginalize underserved populations, where data are limited, thereby deepening health inequalities. We conclude that improving population health and reducing disparities requires reframing prevention through both ethical and economic lenses. Investing in community-based, equity-oriented interventions can deliver sustained health and economic gains and support a more inclusive and resilient health system.

## Introduction

Internationally, policymakers are grappling with how to improve and protect health in the face of complex and urgent national and global challenges. In England, the Darzi Report^1^ highlights the impact of austerity and budgetary cuts to prevention on the state of the nation’s health, creating unsustainable pressure on the taxpayer-funded National Health Service. Simultaneously economic inactivity due to illness has reached record levels.^2^ In short, we cannot afford the current state of ill health. Academics, economists and public health leaders have repeatedly presented evidence of cost effective prevention interventions.^3^ The last decade has however seen progressive disinvestment in prevention rather than proportionate resourcing, with the public health grant cut by 28% on a real terms per person basis since 2015/16.^4^ Further, as life expectancy and healthy life expectancy have stalled or worsened,^5^ cuts to the public health grant have tended to be greater in more deprived areas.^4^

The UK government health mission promises ‘change so we focus on prevention’, delivered through cross-government working and partnership with communities.^6^ The 10 Year Health Plan for England aims to deliver transformational change by moving care into the community and shifting towards prevention, empowering patients and populations.^7^ In the UK, health is devolved, and this discussion focuses primarily on health policy in England. However, prevention cannot be delivered in the healthcare sector alone, and change will require prioritizing core public health objectives and scarce resources across government functions. While this analysis is rooted in the England context, the issues are relevant internationally. We present the case for prevention across population groups and explore how economic arguments help and hinder those aiming to realize the benefits of prevention for populations and communities.

### Using health economics to support public health resourcing decisions

Briefly, cost is an amount that must be spent to buy or obtain a resource input. Once spent, that resource is not available to spend in other ways. A benefit is an advantage, or the value gained from investing in or allocating resources to obtain outputs. Standard economic arguments apply monetary values to the costs and benefits of options for resource allocation, to determine net gains. A cost–benefit ratio estimates the full economic benefit (or lack thereof) of a choice among alternative investments, so society or funders can decide whether investing, for example in health interventions, is a good idea from these perspectives. This type of economic framing puts the emphasis on the inputs and outputs of health interventions and how they relate to each other, rather than the value the investment generates by having impact on the population. For example, comparing the cost–benefit of obesity medications against national policy on the food environment in and around schools can tell us which offer better health and economic returns on investment, but does not capture the broader population impacts. These might include mental wellbeing and future employment for children in deprived communities which are not represented through changes in obesity prevalence, and indirect effects such as reactive changes in marketing strategies by restaurants and food companies. For those aiming to realize benefits for populations and communities cost–benefit ratios do not work. Our premise backed up by Naci *et al.*^8^ is that measurement and prioritization of population health impact should underlie economic arguments.

### The economics of public health and prevention for population health

A pressured fiscal climate has driven short-term budgets and planning cycles, which incentivize short-term perspectives on health. Health service demand is easily measurable, compared to wider population-level direct and indirect impacts. Population health and wealth are intrinsically linked, and approaches such as Return on Investment (ROI) aim to capture wider benefits and costs of health interventions. The ROI for public health interventions are significant, from £4 to 27 per £1 invested.^9^ However, this risks public health interventions being expected to generate cost savings,^10^ primarily for short term budgets in the healthcare sector. This is in direct conflict with the cost effectiveness thresholds used for healthcare treatments, and the Cost per Quality-Adjusted Life Year (QALY) threshold by NICE for England and Wales which sits between £20,000 and £30,000.^3^ Public health interventions deliver health returns over variable time periods, some within months, but generally accumulating over years.^9^ As resources for healthcare are finite, shifting to prevention will require innovative, integrated funding, alongside decision-makers reconciling how we prioritize health.

The UK economy depends on a healthy, productive population. The Tobacco and Vapes Bill is predicted to bring significant population health benefits and economic returns, as smoking remains the leading cause of preventable death and ill health. Financial costs and benefits for the economy and health system are routinely used alongside the projected health benefits.^11^ This is useful for decision-makers to realize the scale of potential benefits. The 2007 Smoke-free Regulations delivered population-level preventative interventions with an immediate 2.4% reduction in emergency admissions for myocardial infarction,^11^ strong evidence of the power that prevention has to enable greater health.

At population level prevention measures should reduce health inequalities. However, as capacity to benefit from prevention measures varies across subgroups of the population there may be a risk of widening inequalities if the context and levers of prevention are not carefully considered. At system, place and neighbourhood level, measuring economic returns and attributing causality can become increasingly difficult. Whilst it is imperative to monitor effectiveness, a heavy emphasis on economic returns will stifle local innovation to invest in contextualized prevention designed to reduce local inequalities.

In summary, health economics, and particularly ROI, are powerful approaches to assessing health interventions that seemingly offer simple answers. It is important to realize the complexity and limitations of the methodologies in contextualization, certainty and inclusion of cross-sectoral benefits and costs. Reorienting towards prevention requires decision-makers to reconcile different expectations and standards for primary, secondary, and tertiary preventative and treatment-based health interventions, and focus on health across the population as a building block for local and national economies.

### Ethics and community engagement in public health decision making

These conflicting approaches to valuing health interventions and population impact also raise ethical concerns about societal values in public health decision making.

In recent years, there has been a renewed focus on the importance of values and societal perspectives in shaping public health decision making. Notable examples include in the ethical complexity in balancing human rights against infection control when restricting care homes visitors during the COVID-19 pandemic^12^ and the tension between autonomy and protection of the vulnerable in the Assisted Dying Bill debate.^13^ As well of the economic benefit framing seen in the Tobacco and Vapes bill debate, polarized views on freedom emerged, where freedom to choose to buy cigarettes is pitted against diminished freedom caused by tobacco addiction.^14^

These examples illustrate that the mantra of following the science, as a common rhetoric used particularly during the COVID-19 pandemic,^15^ cannot yield answers on which public health interventions to implement when these involve conflicting values. Yet, the value-based perspective seems notable by its absence from the health prevention discussion, and we need further discussion about how this might reflect societal values and different understandings of social justice. Unlike health economics, which public health decision-making often relies on, our experience indicates limited collaboration between ethicists and public health professionals in addressing investment for prevention using a broader systems perspective.

To make progress here also involves understanding of public values. When looking at perspectives on healthcare resource allocation, there is evidence of a degree of societal willingness to sacrifice some degree of potential QALY gains to promote equity in how these QALYs are distributed.^16^ This gives credibility to the perspective that societal values are more than about maximizing health benefit, but can include equitable distribution of costs and benefits across different population groups. Nonetheless, care must also be taken in how public opinion contributes to policy, especially around prevention. There can be large differences between what people think is important for good health, and what drives good health, particularly around the overestimation of the importance of healthcare and underestimation of social determinants of health.^17^ Conversely, engaging civil society on how industries like tobacco, ultra-processed food, fossil fuel and alcohol impact health is an important strategy for addressing commercial determinants of health through governmental and societal action. Finally, whilst seeking more input into public values it is important to recognize the power imbalance between policymakers and marginalized communities, shaped by historic and ongoing inequalities. Engagement with the public through methods such as deliberative democracy, a research method which facilitates in depth reflection on preferences, values and relevant considerations, can provide valuable contributions to these nuanced problems.^18^

Implementing this broader understanding of health values can occur through existing structures, designed to put budget and decision making into the hands of local partnerships who will work with local communities to meet local needs. These ambitions are bolstered by progress during COVID-19 in working with communities and building shared decision-making through integrated neighbourhood teams.^19^ Considering how public health interventions can be locally led, cost-effective, and value-driven is an ethical imperative.

### Bringing together health economics and ethics for health inequalities

Reducing health inequalities and strengthening health equity are core priorities for improving public health, and the Marmot Review makes a clear case that measures of health and wellbeing across societal groups are as important as economic growth.^5^ The CORE20PLUS5 evidence-based framework to reduce health inequalities guides health system leaders in England in their health and wellbeing strategic decisions.^20^ Applying CORE20PLUS5 requires an evidence-based data-led approach, but in practice the PLUS groups are variably defined, often lacking from data sets and there is a relative paucity of cost-effectiveness evidence for interventions to improve health for some PLUS groups. At a service level, guidance on best practice is available and NICE uses principles of social justice alongside cost-effectiveness.^21^ Nonetheless, over-reliance on health economics can mean decision-makers under-invest in already marginalized groups citing ‘lack of economic evidence’.

The distinction between health and social issues and interventions can also be contradictory and counterproductive. Homelessness is associated with an average age of death of 43 and 47 years, worse than many chronic diseases, and ~30 years earlier than the general population.^22^ Meanwhile, public health interventions that benefit PLUS groups, such as poverty alleviation and social housing, are more often valued in their returns economically than in population health gains. Rising rates of homelessness^23^ are therefore not treated as an urgent health crisis, but solely as a housing problem for the Treasury. In reality, people in PLUS groups often experience multiple disadvantages, including a negative cycle of poverty and ill-health, meaning healthcare needs and treatment-related costs can be high. These issues are further compounded by the stigma commonly experienced by PLUS groups and structural inequalities that may prevent participation alongside other communities. Effective public health interventions cut across sectors, acknowledging that most population health is not determined by the healthcare system. This becomes increasingly important for groups experiencing more severe health inequalities.

Although integrating health economics with population health is advocated for, this positioning is not without risks. Whilst population health is essential for economic wellbeing, short timelines and economic perspectives can mean investment in health incurs an economic loss. Further, some groups may require additional resources to deliver prevention, with the danger that this is represented as an economic burden. This logic can open the door to disinvestment.

A counter to this risk is to frame health as a more fundamental right or need. Venkatapuram (2011),^24^ for instance, argues that the capability for health is essential for human flourishing and cannot be reduced to its economic contribution. Yet such principled arguments may not always resonate with policymakers, who often respond more readily to economic rationales. Our position, therefore, is that while economic arguments can serve as a pragmatic entry point for influencing policy, they must always be underpinned by a value-based perspective that recognizes health as more than an instrument of economic productivity.

Reorienting and committing to achieving better health across population groups as a societal good would bring greater clarity. It would also drive multisectoral coordination, whether across government departments or integrated care partnerships, ultimately generating greater health and wider returns.

### A forward look

We have argued for realigning health-related decisions with societal ethics and community priorities. This requires the appropriate and effective use of health economics and public health ethics to strengthen the case for the benefits of robust public health for society.

An over-emphasis on short term return on investments in public health both leads to inadequate investment in prevention and risks excluding marginalized groups, for whom there is a lack of evidence to ‘invest’ in. When combined with the complex systems that public health interventions operate within, and the need for extended periods for benefits to be realized, it is important that public health does not over rely on health economic arguments to the detriment of equity considerations.

However, the appropriate use of economic evidence in public health should be applauded. This is especially in comparison to the lack of practical collaboration between public health ethics and practice. A public health ethics perspective to examine what and whose values drive decisions about prevention is long overdue.

Everyone working to improve population health should take opportunities within health policy, a strong mandate from the public for change, devolution of decision making and delivery, and learning from previous experience to reset the relationship between public health, economics and evidence. We should prioritize population health outcomes and health inequalities via investing in national and local prevention. Economic gains will flow from this.

## Notes

**Kathryn Hamilton**, Consultant in Public Health Medicine

**Frances C. Butcher**, Academic Clinical Lecturer in Public Health

**Jane Powell**, Professor of Public Health Economics
